# NEDOCS vs subjective evaluation, ¿Is the health personnel of the emergency department aware of its overcrowding?

**Published:** 2017-06-30

**Authors:** Mauricio Garcia-Romero, Claudia Geraldine Rita-Gáfaro, Jairo Quintero-Manzano, Anderson Bermon Angarita

**Affiliations:** 1 Departamento de Urgencias, Fundación Cardiovascular de Colombia, Floridablanca, Colombia.; 2 Centro de Investigaciones de la Fundación Cardiovascular de Colombia, Floridablanca, Colombia.; 3 Estudiante PhD Epidemiología y Bioestadística, Universidad CES, Medellín, Colombia.

**Keywords:** Emergency medical services, crowding, attitude of health personnel, surge capacity, social perception, health services needs and demands

## Abstract

**Introduction:**

An emergency department (ED) is considered to be "overcrowded" when the number of patients exceeds its treatment capacity and it does not have the conditions to meet the needs of the next patient to be treated. This study evaluates overcrowding in the emergency department of a hospital in Colombia.

**Objective:**

To compare the objective NEDOCS scale with a subjective evaluation by ED health staff in order to evaluate the differences between the two.

**Methods:**

The NEDOCS scale was applied and a subjective overcrowding survey was administered to the medical staff and the charge nurse on duty 6 times per day (6:00 a.m., 9:00 a.m., 12:00 p.m., 3:00 p.m., 6:00 p.m. and 9:00 p.m.) for three consecutive weeks. The results were evaluated with a correlation analysis and measurement of agreement.

**Results:**

A median NEDOCS score of 137 was obtained for the total data. There was a moderately positive correlation between the NEDOCS and the subjective scales, with a rho of 0.58 (*p* (0.001). During times when the ED was the most crowded, 87% of the total subjective health staff evaluations underestimated the level of overcrowding.

**Conclusions:**

Health staff do not perceive a risk due to ED overcrowding when the NEDOCS scores correspond to overcrowding categories equal to or over 5 (severely crowded and dangerously crowded), which poses a risk to patient safety and care.

## Resumen

**Introducción:**

Se considera "Aglomeración" cuando un servicio de urgencias excede el número de pacientes que tiene capacidad de atender o no cuenta con las condiciones para cubrir las necesidades del próximo paciente a ser atendido. Este estudio evalúa el sobrecupo del servicio de urgencias en un hospital de Colombia.

**Objetivo:**

Comparar la escala objetiva de NEDOCS con la escala subjetiva del personal de salud en el departamento de urgencias para evaluar la diferencia entre ambas.

**Métodos:**

Se aplicó la escala NEDOCS y una escala subjetiva de sobrecupo en el servicio de urgencias al personal médico y enfermera jefe de turno durante 3 semanas seguidas, 6 veces al día (6:00 a.m., 9:00 a.m., 12:00 p.m., 3:00 p.m., 6:00 p.m., 9:00 p.m.). Se realizaron análisis de correlación y medida de concordancia para evaluar los diferentes resultados.

**Resultados:**

La mediana de NEDOCS para el total de datos fue de 137, se presentó una correlación moderadamente positiva entre la escala NEDOCS objetiva con respecto a la subjetiva Rho 0.58 (*p* <0.001), del total de respuestas en los momentos de mayor congestión el 87% de las valoraciones subjetivas del personal de salud fueron subestimar el nivel de Sobrecupo.

**Conclusiones:**

Cuando los niveles de sobrecupo clasificados por NEDOCS son iguales o superiores a nivel 5 (Severamente congestionado y peligrosamente congestionado) el personal de salud no tiene una percepción del riesgo por sobrecupo del departamento de urgencias, lo que conlleva a un riesgo en la seguridad y atención del paciente.

## Introduction

An emergency department (ED) is considered to be "overcrowded" when the number of patients exceeds its treatment capacity or it does not have the conditions to meet the specific needs of the next patient to be treated ^1^ . Overcrowding in emergency departments creates a risk environment for patients as well as health staff, with evidence of an increase in the untreated demand rate, medication errors and a relative risk of death of 1.34 (CI 95%: 1.04-1.72) after 10 days and 6.1% after 30 days for patients receiving care when emergency departments are overcrowded ^2^ ^-^ ^5^.

Although ED health staff report that overcrowding occurs on a daily basis, this cannot be objectively determined without applying some type of score. Different scales exist for this purpose, such as the NEDOCS (National Emergency Department Overcrowding Study), EDWIN (The Emergency Department Work Index), READI (Real-time Emergency Analysis of Demand Indicators) and EDCS (Emergency Department Crowding Scale) ^6^ ^,^ ^7^. 

Our group of researchers chose to evaluate the NEDOCS scale given experience using it at the national level and because it is considered to be a simple and quick tool to determine the level of crowding at emergency departments. It contains six categories which range from not busy to dangerously overcrowded ^2^ ^,^ ^8^.

The purpose of this study was to objectively measure the level of overcrowding at an emergency department and the correlation of that measurement with the subjective perception of ED staff.

## Material and Methods

This is an observational and prospective study performed at the Cardiovascular Foundation of Colombia, an institution specializing in highly complex cardiac pathologies. The study was conducted between April and May of 2014.

The NEDOCS scale was applied and a subjective survey on overcrowding was administered to medical staff and the charge nurse on duty six times per day (6:00 a.m., 9:00 a.m., 12:00 m., 3:00 p.m., 6:00 p.m. and 9:00 p.m.) for three consecutive weeks. The staff participating in the study had over two years of experience working in the ED.

The calculation of the NEDOCS score included, as fixed values, 9 ED beds and 189 hospital beds for adults and children, reflecting the installed capacity at the institution. The other values used for the scale were: total patients in the ED, total admits in the ED (based on hospital admissions ordered by the medical specialists), number of respirators in the ED, longest admit time and waiting room wait time for the last patient called.

The NEDOCS was calculated using an official webpage ^9^ and the results were interpreted according to the recommendation by the author, as follows: 0-20 not busy, 21-60 busy, 61-100 extremely busy but not overcrowded, 101-140 overcrowded, 141-180 severely overcrowded and 181-200 dangerously overcrowded. 

The survey on the subjective evaluation of ED overcrowding captured the opinions of the physicians and charge nurses about the level of overcrowding in the ED. This was quantified on a scale of 1 to 6 at the same time the NEDOCS score was registered. On the subjective scale, 1 reflected the opinion that the department was not busy, 2 that it was busy, 3 extremely busy but not overcrowded, 4 overcrowded, 5 severely overcrowded and 6 dangerously overcrowded. For comparison purposes, the NEDOCS scores were adjusted to this same range of 1 to 6 ^10^.

A Likert survey was also administered to the physicians which reflected the level of "feeling rushed" or "pressured" with respect to their work at the moment the survey was administered. This also ranged from 1 to 6, where 1 reflected not feeling rushed and 6 represented feeling the most rushed in terms of work or emotionally stressed. This permitted correlating the perception of ED overcrowding with the level of concern or pressure to treat patients. 

The surveys were administered by two students who were in their last year of medical school and were trained to collect the data and administer the surveys.

A descriptive analysis of the variables was performed, with medians of central tendency, dispersion and percentages. This was followed by a bivariate analysis and a Spearman correlation analysis to correlate the differences among the variables of interest. The statistical analyses were performed with Stata^®^ 12.1 software. 

The present study was evaluated by the research ethics committee of the Cardiovascular Foundation of Colombia.

## Results

This study obtained a total of 126 NEDOCS scores, 126 surveys from ED charge nurses and 200 surveys from ED physicians (since there were two physicians on duty at 12:00 p.m., 3:00 p.m. and 6:00 p.m.).

The median NEDOCS for the total data was 137, which corresponds to overcrowding (Table 1).

**Table 1:** Data recorded in the emergency department (ED) for the NEDOCS scale

Variables	Median	Range Q1-Q3	Min	Max
Total patients in the ED	8	7-11	3	20
Total admits in the ED	6	4-7	1	11
Number of respirators in the ED	0	0	0	1
Longest admit time in the ED (hrs)	64.5	40-89	7	134
Waiting room wait time for last patient called (min)	20	15-60	1	4
NEDOCS	137	114-176	34	200

A description of the percentage of agreement between the NEDOCS scale and the subjective ED physicians' scale was generated by category (Table 2). The subjective health staff survey was in 100% agreement with the times when the NEDOCS was categorized as "busy," whereas the physicians considered the ED to be "dangerously overcrowded" only 15.2% of all of the times the NEDOCS score was classified as such.

**Table 2:** Correlation between the subjective and objective scales.

NEDOCS\Subjective physicians' scale	Not busy	Busy	Very busy but not overcrowded	Overcrowded	Seriously overcrowded	Dangerously overcrowded
21-60	0	4	0	0	0	0
(Busy)*	0	100	0	0	0	0
61-100	3	11	4	6	0	0
(Very busy but not overcrowded) *	12.5	45.8	16.7	25.0	0	0
101-140	4	30	35	10	1	0
(Overcrowded) *	5.0	37.5	43.8	12.5	1.3	0
141-180	2	7	18	13	4	2
(Seriously overcrowded) *	4.3	15.2	39.1	28.3	8.7	4.3
181-200	0	2	6	13	18	7
(Dangerously overcrowded)*	0	4.3	13.0	28.3	39.1	15.2
Total	9	54	63	42	23	9

*percentage

**Figure 1:**
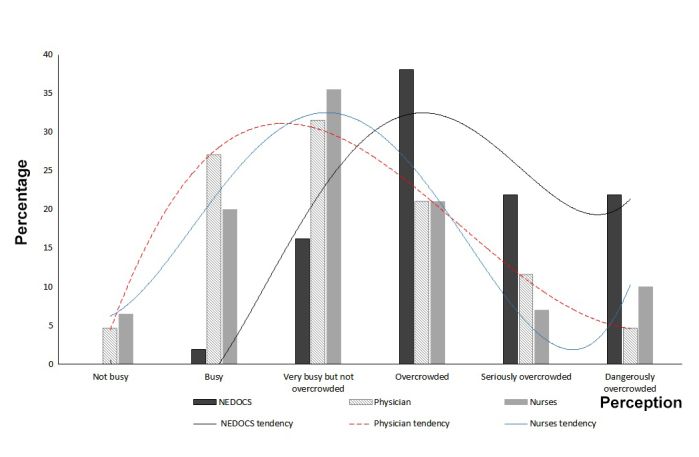
Perception of physicians and nurses of ED overcrowding versus NEDOCS.

Figure 1 shows the differences between the overall perception of the physicians and nurses and the objective scale. The curve representing the subjective scale tends towards normal with a peak at the category "very busy but not overcrowded" while the objective scale's curve leans predominantly to the right, towards greater overcrowding. A positive correlation of 0.58 (*p* ≤0.001) was found between the objective NEDOCS scale and the subjective scale representing all of the health staff.

The comparison between the subjective physicians' scale and the NEDOCS score resulted in a 16.4% agreement with a Kappa of 0.006, which indicates a lack of agreement between the scales. Nurses had a better perception of ED overcrowding than the physicians, with a kappa index of 0.074, which is still very low and lacks agreement (Table 3).

**Table 3:** Kappa index of subjective scales, by profession, and physicians "feeling rushed"

Agreement with the objective NEDOCS	Percentage of agreement	Percentage of Expected agreement	Kappa	Standard error	*Z*
Subjective physician scale	14.50	16.40	-0.0227	0.0280	-0.81
Subjective nurses scale	23.02	16.89	0.0737	0.0362	2.03
Feel Rushed scale for physicians	10.20	11.87	-0.0189	0.0228	-0.83

A significant difference in the NEDOCS scale was found between work days versus holidays (*p*= 0.006, Coef: 21.27 (CI 95%: 6.22-36.33)), with less overcrowding on holidays. There continued to be a lack of agreement between the objective scale versus both the subjective physicians' and the nurses' scales, even after adjusting for work days, while the "feeling rushed" scale better correlated with the objective NEDOCS on holidays.

**Figure 2:**
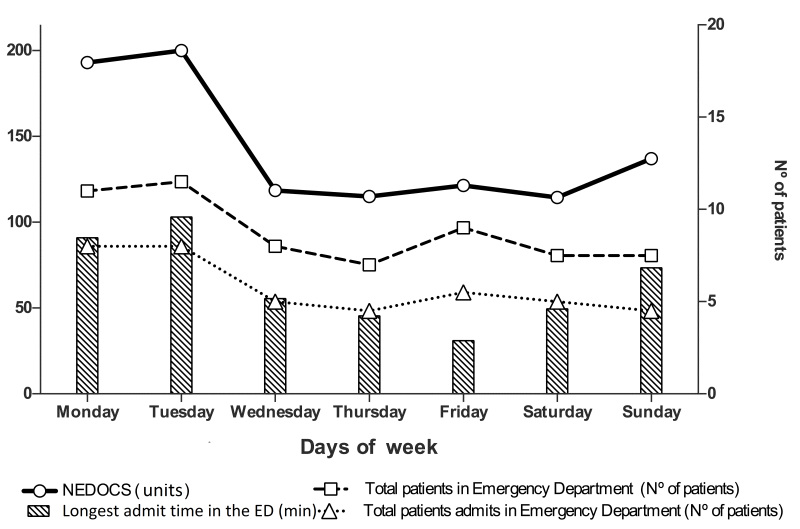
NEDOCS category according to day of the week

A relationship was observed between a high NEDOCS score and Mondays and Tuesdays, due to an increase in the number of patients in the ED, the number of admits and the longest admit time (patients in the ED and admitted were adjusted to the figure by multiplying the value by 10) (Fig. 2).

An association was also found between a high objective NEDOCS scale and the hours 9:00 a.m. (Coef: 18.19, *p*: 0.044; CI 95%: 0.51-35.86) and 3:00 p.m. (Coef: 19.48, *p*: 0.031; CI 95%: 1.8-37.15), adjusted by day of the week and holidays.

With regard to the "feeling rushed" scale, only 13.0% of the physicians reported feeling very rushed during the 46 times when the NEDOCS was 6. The median NEDOCS scale for ED overcrowding was 4 and 78.1% of the time physicians reported a level of 3 or less on the "feeling rushed" scale.

## Discussion

This study shows a significant difference between the NEDOCS score and the health staff's subjective perception of overcrowding in the emergency department, with the staff tending to underestimate the level of overcrowding. In general, no common agreement exists between the subjective perception of health staff and the objective instruments used to measure ED overcrowding ^11^ ^-^ ^13^. One study reported a correlation of k= 0.53 (95% CI: 0.42-0.64) between the NEDOCS quantification of ED overcrowding and the subjective perception of the health team, while another study found a poor correlation between the NEDOCS scale and the health staff's subjective perception (k= 0.31; 95% CI 0.17-0.45) ^2^. Nonetheless neither of these studies presented high NEDOCS scores. The present study did not find a correlation between the health staff's evaluation and the NEDOCS classification of overcrowding even though the scales were evaluated during maximum overcrowding as well as when the ED was not busy.

The values of the subjective physicians' and nurses' scales were not in agreement with the objective NEDOCS scale, particularly when the latter was categorized as 5 or 6 (ranges established by the UNM). The nurses' perception of overcrowding was more similar to the objective scale than the physicians' perception. This may be because nurses are in closer contact with the needs of the ED and with all of the patients, including patients waiting to be treated as well as those waiting to be transferred to a hospital bed. 

One study that compared the NEDOCS scale with health staff perceptions considered the NEDOCS to overestimate ED overcrowding ^15^. Unfortunately, given the design of our study, it is not possible to establish whether the NEDOCS overestimated ED overcrowding or whether the subjective perception of the health staff underestimated it. Nevertheless, it was determined that when the NEDOCS categories were high there was a real limitation on patient flow in the ED, defining the appropriate course of action for patients was slow, and there were delays in treating the patients in the study, thereby increasing patient risks.

This study has the distinctive feature of presenting a high number of times with high NEDOCS scores, with a median of 137, which has not been found by similar studies ^11^ ^,^ ^14^ ^,^ ^16^. This is primarily due to the high volume of demand for emergency services in Colombia and a lack of emergency centers and opportunities for urgent appointments or outpatient visits. 

Studies have found that attending physicians feel less "rushed" than the nurses when emergency departments are overcrowded ^8^, possibly because the subjective perception under evaluation relates more closely with individual workloads than with the level of ED crowding.^17^ Our study found that the nurses had a higher degree of agreement with the NEDOCS index than the physicians, especially with respect to the higher categories, which may be due to the individual activities performed by the nurses. Unfortunately the degree to which the nurses "felt rushed" was not measured.

Emergency departments have been reported to be less crowded on non-working days ^18^, and this is consistent with the present study which found evidence of fewer patients in the ED on weekends and holidays, although admission time was greater.

A high demand and need for emergency department services is common worldwide ^6^ ^,^ ^16^ ^,^ ^18^, and our work group's hypothesis is that health staff may underestimate the level of overcrowding in the ED, possibly because of their lack of knowledge about the objective definition of overcrowding and the corresponding risks to patient safety. This may be partly explained by a lack of international consensus on the matter ^8^ ^,^ ^11^.

The ED at the institute where this study was performed is often the door to hospitalization for patients with highly complex health conditions. This ED treats an average of 4,962 patients annually with an average of two physicians per shift, which may explain the prolonged treatment times and high bed occupancy in both the ED and the hospital.

Some of the factors that affect overcrowding cannot be controlled by the ED staff, for example, transfer time from the ED to a hospital room has been shown to be affected by the institution's hospital occupancy level ^19^. A limitation of our study was that it did not identify specific points that led to delays in transferring patients to hospital services.

Another limitation was using only one health center for the data collection, which makes it difficult to extrapolate the results. Future studies based on more health centers, a larger sample size and longer follow-up periods are expected to be helpful for reaching more definitive conclusions.

At the time of this study, all the physician and nursing staff had worked at the medium- to high-complexity ED for 2 to 8 years, but this data was not included in the subjective evaluation, which also reflects a limitation of the study.

The use of an objective scale which did not depend on individual perception made it possible to evaluate the level of ED overcrowding. Although the adoption of a scale alone clearly does not solve overcrowding problems, it does help to make the medical and administrative staff aware of the need to implement corrective measures based on the needs of each institution, so as to improve the quality of the services they provide and guarantee the safety of both the patients and the health teams.

There is a need for complementary studies that demonstrate the points that need to be improved so that overcrowding in emergency departments can be decreased. 

## Conclusion

When emergency departments are overcrowded, health staff may underestimate patient risk caused by delays or inefficiencies in providing care.
