# Supplementary material for: NEDOCS vs subjective evaluation, ¿Is the health personnel of the emergency department aware of its overcrowding?
Source: Colomb Med (Cali). 2017 Jun 30;48(2):53–7. (PMC5597093)
Supplement: Translation

# Translation: Escala NEDOCS vs valoración subjetiva, ¿El personal de salud en urgencias es consciente de su sobrecupo?

## Introducción

Se considera "sobrecupo" cuando un servicio de urgencias excede el número de pacientes que tiene capacidad de atender o no cuenta con las condiciones para cubrir las necesidades puntuales del próximo paciente en ser atendido ^1^. La congestión en el servicio de urgencias genera un entorno de riesgo tanto para los pacientes como para el personal de salud, con evidencia de aumento en la tasa de demanda no atendida, errores en la medicación, riesgo relativo de muerte a 10 días de 1.34 (IC 95%: 1.04-1.72) y de 6.1% a 30 días en pacientes atendidos durante el sobrecupo en el servicio de urgencias ^2^^-^^5^.

En algunos centros de atención de urgencias el personal de salud manifiesta que el sobrecupo se presenta de manera diaria, sin embargo, no es posible dar un valor objetivo de este sobrecupo a menos que se aplique algún tipo de puntaje, para esto existen diferentes escalas, como NEDOCS (National Emergency Department Overcrowding Study), EDWIN (The Emergency Department Work Index), READI (Real-time Emergency Analysis of Demand Indicators) y EDCS (Emergency Department Crowding Scale) ^6^^-^^7^.

Nuestro grupo de investigación evaluó la escala NEDOCS debido a la experiencia del uso de esta a nivel nacional y por considerarla una herramienta simple y rápida para determinar el grado de congestión en el servicio de urgencias enmarcado en seis categorías que van desde no ocupado hasta peligrosamente congestionado ^2^^,^^8^.

El propósito de este estudio fue medir objetivamente el grado de congestión de un servicio de urgencias, así como su correlación con la percepción subjetiva del personal que labora en dicho servicio.

## Materiales y Métodos

Estudio observacional, prospectivo, realizado en la Fundación Cardiovascular de Colombia, institución especializada en patologías cardiacas de alta complejidad, entre abril y mayo del 2014.

Se aplicó la escala NEDOCS y una escala subjetiva sobre el nivel de sobrecupo del servicio de urgencias al personal médico y enfermera jefe de turno durante tres semanas seguidas, seis veces al día (6:00 y 9:00 a.m., 12:00 m., 3:00, 6:00 y 9:00 p.m.). Este personal contaba con más de dos años de experiencia en el servicio.

Para el cálculo del puntaje de NEDOCS se tomaron como valores fijos 9 camas del servicio de urgencias y 189 camas hospitalarias para adultos y niños lo cual corresponde a la capacidad instalada de la institución. Los otros valores tenidos en cuenta para la escala fueron, total de pacientes registrados en el servicio de urgencias, total pacientes hospitalizados en el servicio de urgencias (Teniendo en cuenta la orden de hospitalización del médico especialista), Número de pacientes críticos en urgencias (Pacientes con soporte ventilatorio mecánico), Tiempo más largo de hospitalización en urgencias y Tiempo en sala de espera del último paciente puesto en cama.

El NEDOCS se obtuvo usando una página web oficial para calcularlo ^9^, los resultados fueron interpretados según la recomendación del autor de la siguiente manera: 0-20 no ocupado, 21-60 ocupado, 61-100 muy ocupado, pero no congestionado; 101-140 congestionado, 141-180 gravemente congestionado y 181-200 peligrosamente congestionado.

La encuesta sobre el valor subjetivo de sobrecupo en urgencias tuvo en cuenta la opinión del médico y la enfermera jefe sobre el grado de congestión del servicio de urgencias cuantificándolo de 1 a 6 en el mismo momento en que se registró el puntaje NEDOCS, donde 1 fue considerar que el servicio no se encuentra ocupado, 2 Ocupado, 3 Muy ocupado, pero no congestionado, 4 Congestionado, 5 Gravemente congestionado y 6 Peligrosamente congestionado. Para fines de comparación, las puntuaciones del NEDOCS objetivo se ajustaron a este mismo intervalo de 1 a 6 ^10^.

Adicionalmente se aplicó a los médicos una encuesta tipo Likert de uno a seis para cuantificar el grado de "sentirse apresurado" o "sentirse bajo presión" en su trabajo al momento de diligenciar la encuesta. Calificando con 1 si no sentía ningún grado de presión y 6 si sentía el mayor grado de presión ya sea a nivel emocional o laboral, con el fin de correlacionar la percepción de congestión en urgencias con su nivel de preocupación o presión para la atención de los mismos.

Las encuestas fueron aplicadas por dos estudiantes de último año de medicina, quienes recibieron entrenamiento para la toma de los datos y la realización de las entrevistas.

Se realizó una exploración de variables de manera descriptiva con discriminación de medidas de tendencia central, dispersión y porcentajes, con un posterior análisis bivariado y con un análisis de correlación de Spearman para correlacionar las diferencias entre las variables de interés. Los análisis estadísticos se llevaron a cabo con el software Stata 12.1^®^.

El presente estudio contó con el aval del comité de ética en investigaciones de la Fundación Cardiovascular de Colombia. 

## Resultados

Se obtuvieron un total de 126 puntuaciones de NEDOCS, 126 encuestas a enfermeras jefe del servicio y 200 encuestas a médicos de urgencias (debido a que en los horarios de 12:00 p.m., 3:00 p.m. y 6:00 p.m. se encontraban 2 médicos de turno).

La mediana de NEDOCS para el total de datos fue de 137, lo cual se interpreta como congestionado. (Tabla 1)

**Tabla 1:** Datos registrados en urgencias respecto a la escala NEDOCS.

Variables	Mediana	Rango Q1-Q3	Min	Max
Total pacientes en Urgencias	8	7-11	3	20
Pacientes hospitalizados en Urgencias	6	4-7	1	11
Pacientes críticos	0	0	0	1
Tiempo más prolongado en urgencias (hrs)	64.5	40-89	7	134
Tiempo en sala de espera hasta ser Admitido (min)	20	15-60	1	4
NEDOCS	137	114-176	34	200

Se realizó una descripción del porcentaje de acierto por categoría entre las escalas NEDOCS y la escala subjetiva contestada por los médicos de urgencias (Tabla 2). El 100% de los momentos en que el NEDOCS fue categorizado como "Ocupado" el personal de salud concordó con esta categoría en la encuesta subjetiva. Del total de momentos en los cuales la escala NEDOCS otorgó una calificación de "Peligrosamente Congestionado" solo en 15.2% de estos, los médicos consideraron que el servicio estaba en esta categoría. 

**Tabla 2:** Relación Escala subjetiva con Objetiva.

NEDOCS\Escala subjetiva médicos	No se encuentra ocupado	Ocupado	Muy ocupado pero no congestionado	Congestionado	Gravemente Congestionado	Peligrosamente Congestionado
21-60	0	4	0	0	0	0
(Ocupado)*	0	100	0	0	0	0
61-100	3	11	4	6	0	0
(Muy Ocupado pero no congestionado)*	12.5	45.8	16.7	25.0	0	0
101-140	4	30	35	10	1	0
(Congestionado)*	5.0	37.5	43.8	12.5	1.3	0
141-180	2	7	18	13	4	2
(Gravemente congestionado)*	4.3	15.2	39.1	28.3	8.7	4.3
181-200	0	2	6	13	18	7
(Peligrosamente Congestionado)*	0	4.3	13.0	28.3	39.1	15.2
Total	9	54	63	42	23	9

*Porcentaje

Al tomar la percepción global sobre el servicio de urgencias, tanto de los médicos como de las enfermeras, se observan las diferencias descritas en la Figura 1, donde la percepción subjetiva tiende a una curva de normalidad, con pico en la categoría tres de "Muy ocupado, pero no congestionado", mientras que los valores concernientes a la escala objetiva denotan una curva predominante a la derecha hacia la mayor congestión. Se presenta una correlación positiva de 0.58 (*p* ≤0.001) entre la escala NEDOCS objetiva con respecto a la escala subjetiva presentada por el total de personal asistencial.

**Figura 1:** Percepción de médicos y enfermeras sobre congestión de Servicio de Urgencias vs NEDOCS.

Al comparar la escala Subjetiva de los médicos con el valor NEDOCS se encontró que ambas coincidieron en el 16.4% con Kappa de 0.006, lo que indica que las escalas no concuerdan. Enfermería tiene una mejor percepción de la congestión del servicio al compararse con la de los médicos, con un índice kappa de 0.074, siendo aún muy bajo y no concordante (Tabla 3).

**Tabla 3:** Índice Kappa de escalas subjetivas por profesión y "Sentirse bajo presión" del médico

Concordancia con NEDOCS Objetiva	Porcentaje de acuerdo	Porcentaje de acuerdo esperado	Kappa	Error estándar	*Z*
Escala subjetiva Medico	14.50	16.40	-0.0227	0.028	-0.81
Escala Subjetiva Enfermera	23.02	16.89	0.0737	0.0362	2.03
Escala "Sentirse bajo presión" en médicos	10.20	11.87	-0.0189	0.0228	-0.83

Se encontró una diferencia significativa del NEDOCS entre los días laborales y los festivos (*p*= 0.006, Coef: 21.27 (IC 95%: 6.22-36.33), siendo menor la congestión en los días festivos. La escala subjetiva continuó sin correlación con la objetiva tanto en el grupo médico como en el de enfermería aun ajustando por presencia de día laboral, pero la escala de presión se correlacionó más con el NEDOCS objetivo en días festivos.

Se observó una relación entre el NEDOCS objetivo alto y los días lunes y martes, a razón del aumento en el número de pacientes en urgencias, el número de pacientes hospitalizados y el tiempo más largo en hospitalización (los pacientes de urgencias y hospitalización se ajustaron a la figura multiplicando su valor por 10) (Fig. 2).

**Figura 2:** Categoría de NEDOCS por día de semana.

Además, se evidenció una asociación entre el NEDOCS objetivo elevado y los horarios de 9:00 a.m. (Coef: 18.19, *p*= 0.044; IC 95%: 0.51-35.86) y 3:00 p.m. (Coef: 19.48, *p*= 0.031; IC 95%: 1.8-37.15), ajustado por día de la semana y días festivos.

Respecto a la escala de "sentirse bajo presión" se evidencia que del total de 46 momentos en los cuales el NEDOCS estuvo en 6, solo el 13.0% de los médicos se sintieron bajo mucha presión. La mediana de congestión del servicio por NEDOCS fue de categoría 4 y el 78.1% de los momentos los médicos se sintieron con una presión de 3 o inferior.

## Discusión

Este estudio muestra una diferencia significativa entre el valor NEDOCS y la percepción subjetiva del sobrecupo en el servicio de urgencias por parte del personal de salud quienes tienden a subestimar el nivel de congestión. No existe un común acuerdo respecto a la relación entre la percepción subjetiva del personal de salud y los instrumentos objetivos para medir el sobrecupo en urgencias ^11^^-^^13^. Un estudio evidenció una correlación entre la escala NEDOCS y la percepción subjetiva del equipo de salud para cuantificar el grado de congestión del servicio de urgencias k= 0.53 (IC 95%: 0.42-0.64) ^2^, contrario a esto otro estudio encontró pobre correlación entre la escala NEDOCS y la percepción subjetiva del personal de salud k= 0.31 (IC 95%: 0.17-0.45) ^14^, sin embargo, ninguno de estos estudios presentó niveles elevados de NEDOCS. En nuestro estudio no se evidenció una correlación entre la percepción del personal de salud y el NEDOCS para medir el sobrecupo a pesar de que se evaluaron las escalas tanto en momentos de máximo sobrecupo como en momentos con baja ocupación. 

También se observó que cuando el NEDOCS era de categoría 5 o 6 se presentó una diferencia significativa entre la percepción de enfermería con respecto a la de los médicos de urgencias, y la concordancia es mayor en el grupo de enfermeras que en el de los médicos sobre todo en la categoría 5 y 6, sin conseguir categorías similares a las dadas por la UNM. Esto puede ser debido al mayor contacto que tiene el personal de enfermería con las necesidades del servicio y con el total de pacientes que se encuentran tanto en espera por ser atendidos como en ser derivados a una cama hospitalaria.

En un estudio se consideró que la herramienta NEDOCS sobreestima el estado de congestión de los servicios de urgencias al compararlo con las percepciones del personal de salud ^15^. Lamentablemente por el diseño de nuestro estudio no se puede llegar a establecer si existe una sobre estimación del NEDOCs con respecto al sobrecupo o si la percepción subjetiva de personal asistencial está subestimando el sobrecupo del departamento, lo cierto es que cuando el NEDOCs presentó categorías elevadas había una limitación real de flujo de pacientes por el servicio de urgencias, baja velocidad para definir una conducta para con los pacientes y demora en la atención de los pacientes del estudio, los cuales aumentan los riesgos de los mismos.

Este estudio tiene la particularidad de presentar un alto número de momentos con calificación de escala NEDOCS elevada. Se encontrando una mediana de 137, lo cual no se evidencia en otros trabajos similares ^11^^,^^14^^,^^16^. Este resultado se debe principalmente a los altos volúmenes de solicitud de servicios de urgencias en Colombia y a la falta de centros de urgencias como a la ausencia de oportunidad en consultas prioritarias o consulta externa.

Se ha evidenciado que en los momentos en que el servicio de urgencias se encontró severamente congestionado los médicos de turno se sentían con menos "sentimiento de apresuramiento" que las enfermeras ^8^, posiblemente porque la percepción subjetiva evaluada se relaciona más con la carga de trabajo individual que con el grado de congestión del servicio de urgencias ^17^, nuestro estudio evidenció que las enfermeras debido posiblemente a sus actividades individuales tienen un mayor grado de concordancia con el índice NEDOCS que los médicos sobre todo en las categorías superiores, pero lamentablemente no se midió el nivel de "sentimiento de apresuramiento" en el personal de enfermería.

Se ha considerado que en periodos de días no laborales el servicio de urgencias presenta menos congestión ^18^, lo cual concuerda con el presente estudio donde se evidencia que los fines de semanas o días festivos disminuye la afluencia de pacientes al servicio de urgencias, pero aumenta el tiempo de admisión.

Es frecuente la alta demanda de atención y necesidad de los servicios de urgencias a nivel mundial ^6^^,^^16^^,^^18^ y la hipótesis de nuestro grupo de trabajo es que el personal de salud puede llegar a subestimar el grado de sobrecupo de estos servicios, lo cual puede deberse a la falta de conocimiento por parte de médicos y enfermeras acerca de una definición objetiva de sobrecupo y los riesgos que esto significa para la seguridad del paciente, esto puede ser explicado en parte por la falta de un consenso internacional al respecto ^8^^,^^11^.

Este estudio se realizó en una entidad cuyo servicio de urgencias por lo general es la antesala de hospitalización de pacientes de alto nivel de complejidad, con un promedio de 4,962 atenciones al año y con un promedio de dos médicos por turno, lo cual puede explicar los tiempos prolongados de atención y la alta ocupación de camas tanto en el servicio de urgencias como en hospitalización.

Hay factores que no pueden ser controlados por el personal del servicio de urgencias que influyen en el sobrecupo, se ha demostrado que el tiempo de hospitalización en urgencias es influenciado por el grado de ocupación hospitalaria de la institución ^19^. Fue una limitante de nuestro estudio no identificar los puntos en específico que llevaron a retraso en el traslado de los pacientes a servicios hospitalarios.

Otra limitación fue contar con solo un centro de salud para la captura de datos, lo cual dificulta la extrapolación de los resultados, se esperaría que, en posteriores estudios con mayor tamaño de muestra, mayor seguimiento y diferentes centros de salud faciliten llegar a conclusiones más contundentes.

Al momento del estudio todo el personal médico y asistencial llevaba entre dos y ocho años trabajando en el servicio de urgencia de mediana y alta complejidad, pero fue una limitante no discriminar este dato al evaluar la escala subjetiva. 

El contar con una escala objetiva cuyo valor no dependa de percepciones individuales permite evaluar el nivel de sobrecupo en un servicio de urgencias, es claro que la sola adopción de la escala no soluciona los problemas de sobrecupo en urgencias, pero permite concientizar al personal tanto médico como administrativo de la necesidad de implementar medidas correctivas dependiendo de las necesidades propias de cada institución con el fin de mejorar la calidad de los servicios brindados y garantizar tanto la seguridad del paciente como la del equipo de salud.

Se requieren estudios complementarios para evidenciar los puntos a mejorar que permitan disminuir la aglomeración en los servicios de urgencias.

## Conclusión 

Ante la presencia de aglomeración en los servicios de urgencias se puede presentar subestimación por parte del personal asistencial respecto al riesgo que pueden tener los pacientes por demoras o ineficiencias en la atención.
